# HIV incidence among sexual health clinic attendees in England: First estimates for black African heterosexuals using a biomarker, 2009-2013

**DOI:** 10.1371/journal.pone.0197939

**Published:** 2018-06-20

**Authors:** Adamma Aghaizu, Jennifer Tosswill, Daniela De Angelis, Helen Ward, Gwenda Hughes, Gary Murphy, Valerie Delpech

**Affiliations:** 1 National Infection Service, Public Health England, Colindale Avenue, London, United Kingdom; 2 Department of Infectious Disease Epidemiology, School of Public Health, Imperial College London, Norfolk Place, London, United Kingdom; 3 MRC Biostatistics Unit, Institute of Public Health, Forvie Site, Cambridge, United Kingdom; National and Kapodistrian University of Athens, GREECE

## Abstract

**Introduction:**

The HIV epidemic in England is largely concentrated among heterosexuals who are predominately black African and men who have sex with men (MSM). We present for the first time trends in annual HIV incidence for adults attending sexual health clinics, where 80% of all HIV diagnoses are made.

**Methods:**

We identified newly diagnosed incident HIV using a recent infection testing algorithm (RITA) consisting of a biomarker (AxSYM assay, modified to determine antibody avidity), epidemiological and clinical information. We estimated HIV incidence using the WHO RITA formula for cross-sectional studies, with HIV testing data from sexual health clinics as the denominator.

**Results:**

From 2009 to 2013, each year, between 9,700 and 26,000 black African heterosexuals (of between 161,000 and 231,000 heterosexuals overall) were included in analyses. For the same period, annually between 19,000 and 55,000 MSM were included. Estimates of HIV incidence among black Africans increased slightly (although non-significantly) from 0.15% (95% C.I.0.05%-0.26%) in 2009 to 0.19% (95% C.I.0.04%-0.34%) in 2013 and was 4-5-fold higher than among all heterosexuals among which it remained stable between 0.03% (95% C.I.0.02%-0.05%) and 0.05% (95% C.I.0.03%-0.07%) over the period. Among MSM incidence was highest and increased (non-significantly) from 1.24% (95%C.I 0.96–1.52%) to 1.46% (95% C.I 1.23%-1.70%) after a peak of 1.52% (95%C.I 1.30%-1.75%) in 2012.

**Conclusion:**

These are the first nationwide estimates for trends in HIV incidence among black African and heterosexual populations in England which show black Africans, alongside MSM, remain disproportionately at risk of infection. Although people attending sexual health clinics may not be representative of the general population, nearly half of black Africans and MSM had attended in the previous 5 years. Timely and accurate incidence estimates will be critical in monitoring the impact of the reconfiguration of sexual health services in England, and any prevention programmes such as pre-exposure prophylaxis.

## Introduction

In England, the HIV epidemic is concentrated in two populations, black African men and women who acquired their infection heterosexually, many of whom were born abroad, and men who have sex with men (MSM). New HIV diagnoses, often used as a proxy for monitoring epidemic trends, have remained stable over the last five years at around 6,000 cases each year, down from a peak of over 7,000 in 2005.[1] Among black African heterosexuals, new HIV diagnoses have decreased over the ten year period from 3663 in 2004 to 1113 in 2013 (observed figures) whilst among MSM it increased from 2415 in 2004 to 3,058 in 2013. In parallel, the number of black Africans and MSM testing in sexual health clinics has increased (from 37,701 in 2009 to 46,457 in 2013 and 58,698 in 2009 to 102,553 in 2013, respectively).[2]

Disentangling how much the change in new HIV diagnoses is due to changes in testing or transmission requires accurate estimates of incidence. No estimates of HIV incidence exist for heterosexuals in the UK. National estimates for MSM indicate that incidence has remained stable or increased only slightly over the ten year period. These were derived using models based on serial prevalence estimates [3], back calculations of CD4 cell count data [4] or simulations of risk behaviours [5].

Models for MSM cannot be adapted for heterosexuals since behavioural data are lacking and the assumptions concerning migration are inappropriate, in particular for ethnic minority populations. Although new HIV diagnoses have been decreasing among heterosexuals, mainly due to fewer diagnoses from persons born abroad [1], they still account for approximately half of all new diagnoses. Of note, it is estimated that half of heterosexuals born abroad diagnosed with HIV acquired their infection in the UK.[1]

Many countries are now turning towards the use of biomarkers for recent HIV infection to estimate incidence. These have the potential to produce timely results at relatively low cost.[6–12] Ongoing debate regarding the accuracy of these tests[13,14] prompted a Bill and Melinda Gates funded initiative known as the Consortium for the Evaluation and Performance of HIV incidence Assays (CEPHIA) to evaluate and publish accurate performance characteristics of these assays.[15,16] Public Health England (PHE) first introduced testing for recent HIV infection in sentinel sexual health clinics in 1999 [17] which subsequently developed into a national programme to be offered to all newly HIV diagnosed persons in England in 2009.[18] In England sexual health clinics remain the most important setting for HIV testing for MSM and heterosexual men as well as heterosexual women alongside antenatal testing.[1] About half of all black African men and women and MSM living in the UK have had an HIV test at a sexual health clinic in the past 5 years (46% of black African women, 44% of black African men, 52% of MSM). [19] Thus a significant fraction of new infections is likely to be diagnosed in these clinics providing a highly efficient place to encounter incident infections. This study provides a unique opportunity to examine HIV incidence in sexual health clinic attendees by combining comprehensive testing data from all sexual health clinics with incidence biomarker data. In particular, for the first time, estimates for black African communities and heterosexuals overall are calculated.

## Methods

### Data sources

#### New HIV diagnoses

PHE collates national data on all diagnoses of new HIV, AIDS and AIDS-related deaths along with demographic and epidemiological information for persons aged over 15 years in the UK from clinics and laboratories. Information collected includes sexual orientation, age, gender, ethnicity, country of birth, probable country of infection, place of diagnosis, diagnosis date and HIV type. Information on antiretroviral therapy (ART) and viral load are collected prospectively, annually, through surveys of persons attending clinics for HIV care.[1]

#### HIV testing data

Since 2008, the Genitourinary Medicine Clinic Activity Dataset (GUMCAD) collated at PHE has collected data on all attendances and services delivered at sexual health clinics in England including HIV tests, age, gender, sexual orientation ethnicity and country of birth.[20] In England, all attendees of sexual health clinics should be offered a HIV test and coverage is high (70% of attendees were tested in 2013, 77% of heterosexual men, 66% of women and 86% of MSM).[2]

#### Laboratory methods

Testing for recent HIV infection is performed centrally at PHE. During the study period the AxSYM assay HIV 1/2 gO (Abbott, United States) was used, modified to measure antibody avidity [21], which is typically weaker during the initial stages of infection [6]. An antibody avidity index value of less than 80% was the cut-off for recent infection. Avidity results were linked to new HIV diagnosis reports on sex, date of birth, soundex (scrambled surname code) [22] and diagnosis site [18]

A recent infection testing algorithm (RITA) was used to minimise misclassification; cases classified as recent by the assay were reclassified as long-standing infections if the patient had an AIDS-defining illness within a year of diagnosis, or a CD4 count<50 cells/mm^3^ at diagnosis, or the patient had had prior ART (indicated either by information on treatment or a viral load <400 copies/mL; CD4 and viral load data available for 94% of cases where avidity<80%). We used 181 days as the mean duration of recent infection (MDRI) for the assay (personal communication Daniela De Angelis).

The coverage of testing for recent infection increased from 25% of all new HIV diagnoses in 2009, to 50% in 2013 and we found that those tested for recent infection were broadly representative of all persons newly diagnosed by the demographic variables available; in 2013 the coverage was 52% (1100/2101) among heterosexuals, 60% (1704/2838) among MSM, 56% (1429/2543) among persons aged <35 years, 51%(1582/3087) among those ≥35 years and 58% (1529/2659) in London versus 50% (1483/2975) outside London.

#### The false recent ratio

Currently, all available biomarkers assays misclassify a fraction of longstanding infections as recently acquired.[23] We estimated the proportion false recent, also termed the false recent ratio (FRR), in our population by identifying the number of cases defined by our RITA as recently infected among a subset of patients known to have been diagnosed more than a year previously. These specimens were from patients who had transferred their care from one clinic to another and were therefore not diagnosed for the first time at the latter clinic which sent a specimen for recent infection testing (excluded from the main analyses).

### Statistical methods

All data are fully anonymised at the time of analysis. A serial cross-sectional study design was used to estimate HIV incidence examining the number of incident infections among a population tested for HIV using [24]:

$$lr=\frac{\text{R}-\in \text{P}}{\left(1-\in \right)\text{wN}}$$

Where:

lr = Annual rate

R = the number of recent infection cases

∈= the False Recent Ratio (FRR)

P = the number of HIV positive people tested for recent infection

W = the window period/MDRI

N = the number of people that tested negative for HIV

Biomarker data were used to determine the number of recent infections (R). As not all new diagnoses were tested for recent infection (50% in 2013), we used HIV testing data from GUMCAD to extrapolate a corresponding number of persons tested for HIV each year (T_R_) and number of persons that tested negative (N). In GUMCAD, for each risk group, we calculated the total number of HIV tests per diagnosis (diagnosis rate) and used this as a multiplier for the number of positive cases (P):

$$T_{R}=P_{R}\left(\frac{T_{G}}{P_{G}}\right)$$

Where:

T_R_ = the number of people that tested for HIV (corresponding to P_R_)

P_G_ = the number of HIV positive people observed in GUMCAD

T_G_ = the number of people that tested for HIV in GUMCAD

To obtain the number of negative HIV tests (N), we subtracted the number of positive tests (which here equates to the number of recent infection tests) (P) from the number of HIV tests (*T*_*R)*_. Each clinic attendee was considered only once each year (the first test) despite possible multiple attendances and tests. Patients diagnosed for the first time at a given clinic each year and with no evidence of previous HIV-related care were considered newly diagnosed.

Annual incidence estimates are presented separately for black African heterosexuals, heterosexuals overall and MSM, and separately for those attending clinics in London where half of all new HIV infections are diagnosed.[1]

Confidence intervals were derived using the delta method approximation recommended in the WHO guidance for use of biomarker assays to estimate incidence.[24] Briefly, the 95% C.I. was calculated using the following:

$$I=\pm 1.96xI_{r}C_{v}$$

Where the coefficient of variation (*Cv*) was calculated using:

$$C_{v}=\sqrt{\frac{1}{P}}\left(\frac{N+P}{N}+\frac{\left(P-R\right)R{\left[1+\frac{\varepsilon }{1-\varepsilon }\right]}^{2}}{{\left[R-\frac{\varepsilon }{\left(1-\varepsilon \right)\left(P-R\right)}\right]}^{2}}\right)+\frac{\sigma_{w}^{2}}{w^{2}}+\frac{\sigma_{\varepsilon }^{2}{\left(P-R\right)}^{2}}{{\left(1-\varepsilon \right)}^{4}{\left[R-\frac{\varepsilon }{\left(1-\varepsilon \right)\left(p-R\right)}\right]}^{2}}$$

with

*σ*_*w*_ = the standard deviation of the mean RITA distribution

*σ*_*ε*_ = the standard deviation of the FRR

Inferences of trends over time were made based on the width of the variances around the annual estimates.

All data were managed and analysed in Stata 13.0 (Stata Statistical Software: Release 13, United States).

## Results

### Number of HIV tests, diagnoses and the proportion of recent infection

For each year between 2009 and 2013, 144 (of 206), 141 (of 206), 136 (of 209), 150 (of 209) and 125 (of 222) sexual health clinics in England submitted specimens for recent infection testing, representing between 56% and 72% of all sexual health clinics in England (2013 data only until September 1st after which a different assay was used). A total of 19,008 new HIV diagnoses were reported by participating clinics, with similar numbers each year (Table 1). The annual number of HIV tests per diagnosis increased from 162 in 2009 to 215 in 2013 and was much higher among heterosexuals overall (increasing from 236 in 2009 to 424 in 2013) compared to black African heterosexuals (increasing from 22.1 in 2009 to 55.0 in 2013) and MSM (increasing from 26.3 in 2009 to 41.4 in 2013). The decrease in diagnosis rate coincides with an increase in HIV tests among the participating clinics (Table 1).

**Table 1:** Estimated annual HIV incidence in sexual health clinics in England; by transmission risk group 2009–2013.

Risk group		All attendees					Heterosexuals												MSM		
							All					Black Africans									
Year		2009	2010	2011	2012	2013*	2009	2010	2011	2012	2013*	2009	2010	2011	2012	2013*	2009	2010	2011	2012	2013*
GUMCAD	N tests taken^a^	699487	694800	739446	774212	520240	518494	561970	633006	667166	447302	23813	26613	29178	33031	23113	44634	51403	65443	71152	53053
	N new diagnoses^b^	4328	4117	4250	3889	2424	2197	1947	2045	1795	1056	1076	961	953	742	420	1698	1636	2010	1941	1280
	Tests per diagnosis^c^	161.6	168.8	174	199.1	214.6	236	288.6	309.5	371.7	423.6	22.1	27.7	30.6	44.5	55	26.3	31.4	32.6	36.7	41.4
	N tested^d^	1478	2230	2724	2700	1665	681	1083	1180	1050	546	440	660	671	585	256	715	997	1428	1497	970
RITA	N avidity <80% ^e^	217	337	483	593	400	64	90	112	114	77	22	39	41	44	26	145	233	356	452	307
	N recent applying RITA^f^	173	286	426	507	353	49	68	89	81	56	16	28	30	29	16	117	206	327	404	283
	% recent^g^	11.7%	12.8%	15.6%	18.8%	21.2%	7.2%	6.3%	7.5%	7.7%	10.3%	3.6%	4.2%	4.5%	4.96%	6.3%	16.4%	20.7%	22.9%	27%	29.2%
	N recent after FRR applied^h^	144.9	243.6	374.2	455.7	321.4	36.1	47.4	66.6	61	45.6	7.7	15.5	17.3	17.9	11.1	103.4	187.1	299.9	375.6	264.6
	% recent after FRR^i^^**^	9.8%	10.9%	13.7%	16.9%	19.3%	5.3%	4.4%	5.6%	5.8%	8.36%	1.7%	2.3%	2.6%	3.1%	4.4%	14.5%	18.7%	21.0%	25.1%	27.3%
Estimated	N tests taken for RITA^j^	238873	376343	473941	537509	357343	160717	312590	365255	390264	231275	9738	18277	20544	26042	14088	18795	31326	46494	54876	40204
	N negative tests^k^	237395	374113	471217	534809	355678	160036	311507	364075	389214	231275	9298	17617	19873	25457	14088	18080	30329	45066	53379	39234
	Estimated incidence^l^ (95% C.I)	**0.13%** (0.10%-0.16%)	**0.14%** (0.12%-0.17%)	**0.17%** (0.15%-0.20%)	**0.19%** (0.16%-0.21%)	**0.20%** (0.17%-0.23%)	**0.05%** (0.03%-0.07%)	**0.03%** (0.02%-0.05%)	**0.04%** (0.03%-0.05%)	**0.03%** (0.02%-0.05%)	**0.04%** (0.03%-0.06%)	**0.18%** (0.03%-0.39%)	**0.19%** (0.04%-0.34%)	**0.19%** (0.05%-0.33%)	**0.15%** (0.05%-0.26%)	**0.17%** (0.05%-0.30%)	**1.24%** (0.96%-1.52%	**1.34%** (1.10%-1.58%)	**1.44%** (1.22%-1.67%)	**1.52%** (1.30%-1.75%)	**1.46%** (1.23%-1.70%)

^a^ data from GUMCAD,

^b^ = data from New HIV Surveillance,

^c^ = a/b,

^d,e,f^ = data from the recent HIV infection testing programme after applying the RITA algorithm

^g^ = f/d,

^h^ = f-(FRR*d),

^i^ = h/d

^j^ = c*d,

^k^ = j-d,

^l^applying the WHO formula^24^

*until August 31^st^ (different assay used after this date)

** False Recent Rate = 1.9%

After applying the RITA algorithm, the proportion of recent infection among samples tested was 9.8% (145/1478) in 2009, increasing to 19.3% (321/1665) in 2013. The proportion of recent infection differed by risk group and increased in all sub-groups over this period; among black African heterosexuals from 1.7% (8/440) to 4.4% (11/256); among heterosexuals overall from 5.3% (36/681) to 8.4% (46/546) and MSM, among whom it was highest, from 14.5% (103/715) to 27.3% (265/970) (Table 1).

### The false recent ratio (FRR)

Of 580 available specimens from persons known to have had an infection for more than a year (not included in the main analyses), 38 were classified by the assay as recent (avidity index<80%). Of these, 27 were correctly reclassified by the RITA algorithm (24 had evidence of prior ART, an additional two had a viral load <400/copies/mL and one had AIDS within a year) yielding a 1.9% (11/580) (95% C.I. 1.0%-3.4%) FRR.

### Estimated HIV incidence among sexual health clinic attendees by risk group and age

**Fig 1:**
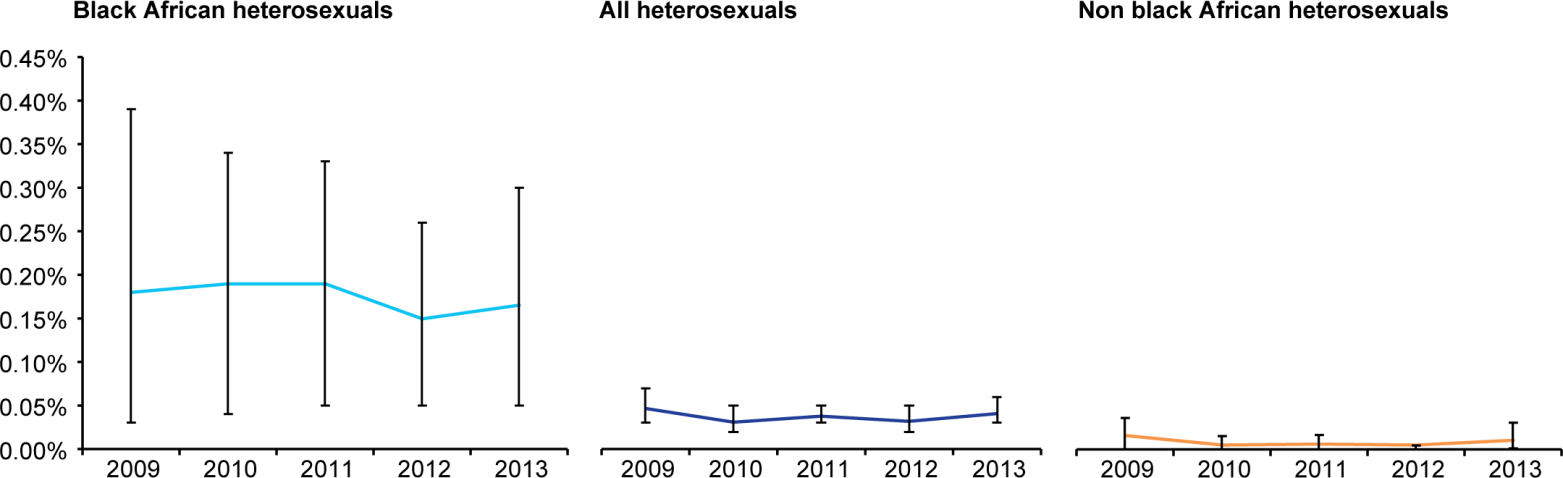
Trends in annual HIV incidence among heterosexual sexual health clinic attendees in England 2009–2013.

**Fig 2:**
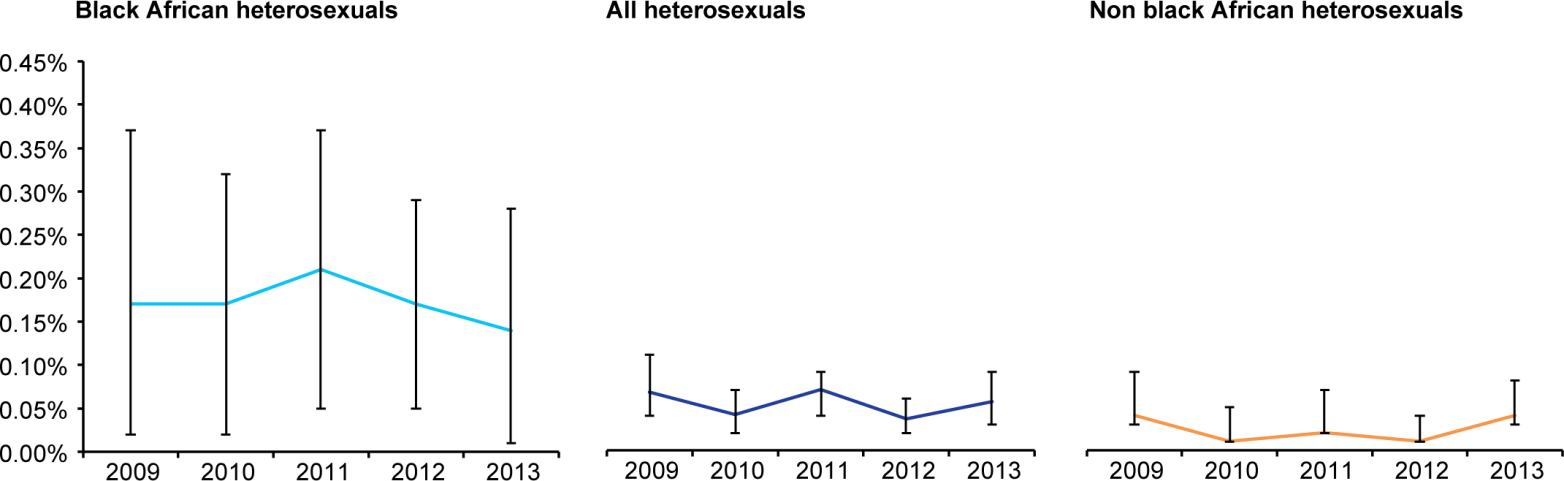
Trends in annual HIV incidence among heterosexual sexual health clinic attendees in London, 2009–2013.

Using an MDRI of 181 days, we estimated annual HIV incidence among sexual health clinic attendees to have increased from 0.13% (95%C.I 0.10%-0.16%) in 2009 to 0.20% (95% C.I. 0.17%-0.23%) in 2013. Annual incidence increased slightly but non-significantly from 0.15% in 2009 (95% C.I 0.05%-0.26%) to 0.19% in 2013 (95% C.I. 0.05%-0.33%) among black Africans and was approximately 4–5 times higher each year compared to heterosexuals overall, among whom it was stable over the period at between 0.03% (95% C.I. 0.02%-0.05%) and 0.05% (95% C.I. 0.03%-0.07%) (Fig 1). For heterosexuals, we examined these data by gender and country of birth however the number of recent infection cases in these sub populations was extremely small resulting in very wide and unstable variance estimates. In London, where approximately 30–40% of HIV tests were undertaken each year (219614 (31%), 232398 (33%), 244525 (33%), 270491 (35%) and 193875 (37%) in 2009, 2010, 2011, 2012 and 2013, respectively), HIV incidence was broadly similar to the rest of England among black Africans and heterosexuals overall (Fig 2).

**Fig 3:**
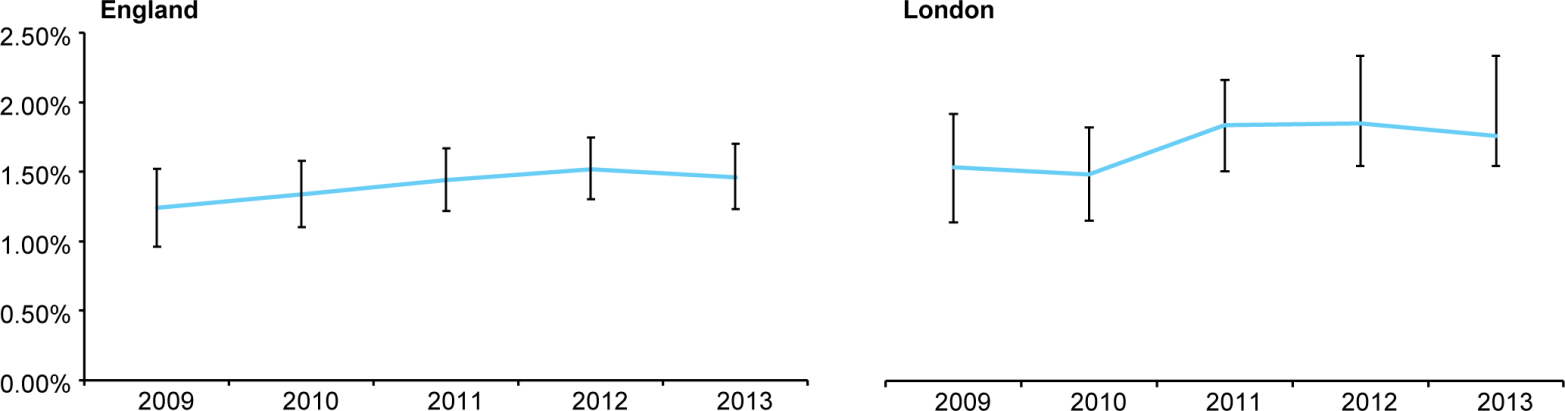
Trends in annual HIV incidence among MSM sexual health clinic attendees by region 2009–2013.

**Fig 4:**
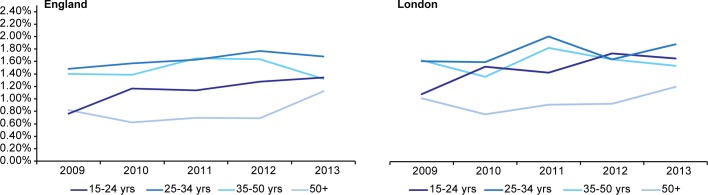
Trends in annual HIV incidence among MSM sexual health clinic attendees by region and age 2009–2013.

HIV incidence among MSM was highest and rose non-significantly from 1.24% (95%C.I. 0.96%-1.52%) to 1.46% (95% C.I. 1.23%-1.70%) (Fig 3). Among MSM in London, estimates were slightly but not significantly higher. Analysis by age showed little difference in incidence by age among heterosexuals (data not shown due to small numbers and consequent unstable variances); among MSM the increase in incidence occurred in all age groups with highest rates among those aged 25–34 years followed by those aged 35–50 years (Fig 4).

## Conclusion

### Principal findings

This is the first study to provide estimates of annual HIV incidence among a representative sample of sexual health clinic attendees and importantly, the first estimates among heterosexual men and women of black African ethnicity, one of the largest sub-populations living with HIV in the UK. The study uses unique methodology combining information from new diagnoses case reports, recent infection biomarkers and HIV testing in sexual health clinics with minimal sampling biases in demographics. We estimate 2 per 1,000 incident HIV cases among all sexual health clinic attendees annually in 2013. Incidence was 1.7 per 1000 for black African heterosexuals, approximately four-fold higher than the 0.4 per 1000 in heterosexuals overall. HIV incidence was highest among MSM at 14.6 per 1000.

The findings suggest a slight increase in annual HIV incidence among MSM over the period, although this was not statistically significant. Of note is that the variance estimates here may be slightly wider than they would be if no RITA data had been missing as the calculations here are based on a smaller sample. The HIV diagnosis rate and the proportion of recent infection diagnosed ranged widely between risk groups reflecting both the variation in testing patterns and underlying HIV prevalence and incidence in these populations. The much lower proportion of recent infection among black Africans may also be due to a larger fraction having likely acquired their infection prior to arriving to the UK.

Our approach provides estimates with improved precision compared to other estimation methods [2–4], because here, the HIV status is known for the whole population of study. This approach is particularly effective for estimating incidence among all risk groups allowing comparison. Using a biomarker circumvents the need for sexual behavioural and migration data as identifying incident infection is based on testing specimens in real time in conjunction with routinely collected clinical data such as viral load and CD4 cell counts.

### Comparisons with other studies

There are no previous studies which have attempted to estimate HIV incidence for the black African community in England or indeed heterosexual populations whilst several estimates exist for MSM. A previous study using biomarkers in sentinel sites among MSM participants of an unlinked anonymous HIV prevalence serosurvey in England and Wales between 1995 and 2005, found a higher rate of HIV incidence (2.45% in 2001 based on 317 recent infection diagnoses).[25] However in this study, incidence is likely to have been overestimated as the assay specific FRR was not accounted for and likely longstanding specimens were not excluded as part of an algorithm. Further, comparing findings is difficult as a different assay was used.

More recent studies estimated HIV incidence among MSM in the general population at national level, one based on back calculations of HIV diagnoses and CD4 cell count [4] and the other on simulations of risk behaviours [5]; Birrel et al’s model estimated 2,300–2,500 new HIV infections among MSM in 2010, which would equate to an incidence rate of 0.4%. Similarly, a stochastic model found from 2006–2010 HIV incidence was 0.53 per 100 person years. [5] A Bayesian evidence synthesis model found that HIV incidence was between 0.5% and 1% among MSM in 2007.[3] Annual incidence estimates among MSM attending sexual health clinics in this study were approximately three-fold higher than estimates among all MSM confirming that sexual health clinic attendees are a higher risk group of HIV acquisition and likely to benefit from HIV prevention initiatives such as pre-exposure prophylaxis (PrEP).

The lack of population-based estimates for black Africans in the literature makes such a comparison difficult and hence we are unable to assess the potential bias among the sexual health clinic attendees of this population.

### Limitations

Epidemiological data from sexual health clinics are influenced by population-level testing patterns creating sampling bias, due to the non-random nature of attendance. For example, patients with a seroconversion illness may be more likely to present for HIV testing, termed the ‘seroconversion effect’. [26] Test-seeking behaviours may also have changed over time due to changes in national HIV testing recommendations; currently, recommendations are for black Africans to test for HIV and to test after every new partner; for MSM they are to test annually or more frequently, e.g. also after every new sexual partner.[1] Motivated and frequent testers have a higher probability of being diagnosed during the earlier stages of infection, therefore potentially inflating the estimated incidence. We observed a large increase in testing over the five year period which likely identified more recent infections, but potentially diluted our incidence estimates due to more low risk persons testing, as demonstrated by the increasing number of tests per diagnosis.

Secondly, sampling bias may occur due to incomplete coverage of recent infection testing (approximately half of new HIV diagnoses). Clinicians could also be more inclined to send specimens for recent infection testing from patients who report a recent risk exposure. However, we previously explored the representativeness of persons tested for recent infection comparing them to all persons newly diagnosed and found no differences in age, ethnicity or country of birth.[18] Although recent infection is associated with some demographic characteristics [18], we acknowledge that similar demographic characteristics may not mean similar risk. In addition the width of the variance estimates is likely to have been overestimated contributing to the difficulty in determining trends over time.

Thirdly, clinical data for the algorithm were not available for 6% of cases with avidity<80%. This may imply the number of recent cases and incidence is overestimated. However we consider our data on treatment status to be complete, capturing most cases with low viral load or CD4 count.

Fourthly, certain patient characteristics may affect the performance of the assay; for example it is known that the HIV subtype affects the FRR [16], and that the MDRI may vary for different population groups (the estimate used here was measured only among MSM). We believe HIV subtype variation is unlikely to have a huge effect on our estimates as the composition of our population regarding transmission risk was similar to the specimens used to calculate the FRR (45% MSM, 49% heterosexuals, 6% other e.g. PWIDs or not reported; see section on FRR). To note, subtype B is mostly diagnosed in the UK (40% overall, 89% among MSM), followed by subtype C (34%).[27] Among heterosexuals subtype C is the most common (50.6%) followed by B (14.8%).[27] Data for the FRR by subtype for other incidence assays show the FRRs for subtypes B and C to be similar for the Limited Antigen Assay ((SEDIA BioSciences) (0.5% FRR for subtype B and 1.3% for subtype C) but vary more significantly for other assays.[16] No such data are available for the assay used here which is no longer commercially available. We undertook sensitivity analyses around the FRR and MDRI and found that increasing the FRR by 1%, from 1.9% to 2.9% or adding 40 days to the MDRI, increasing it by 20% from 181 to 221 days, or both, did not result in estimates outside the variance of those presented here. However, to note is that our FRR is based on diverse subtypes and the MDRI on subtype B, which may imply the FRR estimate is higher than appropriate for the MSM group. This would result in an underestimate of incidence in this subpopulation.

Lastly, patient records in GUMCAD can only be uniquely linked within and not between clinics. Therefore a patient could be coded as newly diagnosed in more than one clinic which would underestimate the number of HIV tests per diagnosis and inflate incidence estimates. Currently, it is estimated that approximately 10% of patients use multiple clinics, of which a subset may be newly diagnosed patients.

### Implications

Testing for recent HIV infection enabled these first estimates for HIV incidence among black Africans among a representative sample of sexual health clinics and showed they remain disproportionately at risk of infection in England. To note is that the sexual health clinic attending population is unlikely to be representative of the general population. However, in this setting, for all population groups, HIV incidence has not decreased over the last half decade despite ongoing prevention and HIV testing initiatives.[28] Interpreting the trend (or lack of) in incidence is difficult as, this may be influenced by an insufficient sample size given the number of new infections.

This may call into question the value of incidence tests; although we have been able to derive incidence estimates with considerable precision, only large changes in incidence over time can be reliably observed due to sample size restrictions and the background prevalence of HIV. A group at the South African Centre for Epidemiological Modelling and Analysis (SACEMA) have published a tool which countries can use to determine the sample sizes required to detect a reduction in incidence over two time points.[29] Using this tool and the characteristics of our assay, estimated incidence and number of HIV tests, we would need to observe a change in incidence greater than 20% to correctly infer a reduction to 5% significance and 80% power (assuming 5% prevalence among MSM and 1% among all attendees). In the absence of any drastic interventions, a reduction to this extent is only likely to be observed over an extended period.

Further, use of antiretrovirals including PrEP can compromise biomarkers [16] However, patients taking PrEP are encouraged to test for HIV every three months at a sexual health clinic and seroconverters will therefore be detected through the genitourinary surveillance system without the need for a biomarker. [30,31]

Our findings shed light on the rates of new infections in black Africans and compare these across sub-populations in the UK for the first time, which is timely, given the recent reconfiguration of sexual health services in the England. In 2013, the commissioning of sexual health services in England was split from HIV care; sexual health and sexually transmitted infection prevention services are assigned to local authorities and HIV treatment and care is funded nationally by NHS England. In addition, providers are moving towards online models for service provision (https://www.sh24.org.uk/) which will result in fewer sexual health clinics and the expansion of home sampling and home testing. As of current, the National Institute for Health and Care Excellence (NICE) guidance recommends HIV testing to be routinely offered to all sexual health clinic attendees, antenatal services, termination of pregnancy services and drug dependency programmes and expanded testing is to be undertaken in areas where more than 2 in 1000 population have been diagnosed with HIV. Our data show a significant scale up of HIV testing in recent years, up to doubling among MSM attending sexual health clinics. With the number of HIV tests required per HIV diagnosis among key risk groups (black Africans and MSM) approximately a tenth that of other heterosexuals (~1 in 40 compared to 1 in 400 in 2012) targeted HIV testing remains efficient and cost effective. This is further supported by the observed stable HIV incidence over the period despite increased testing. It will be crucial to monitor the impact of structural changes to the delivery of sexual health services on HIV testing rates among different sub-populations and importantly, HIV incidence among these groups.

With black African men and women most likely to be diagnosed late [1] more work is needed to improve HIV testing rates in this group and promote regular testing. Stigma remains a huge barrier towards testing and successful prevention efforts in this population.[32] Although a large proportion of infections are acquired in the UK, the Mayisha II study showed nearly half of black African men and women travelled to their country of origin in the previous five years and 40% of men and 22% of women acquired a new sexual partner when abroad.[33] An association between travelling to their country of origin and high sexual risk such as larger numbers of partners and history of a sexually transmitted infection diagnosis was also one of the findings. More work is needed to develop innovative, targeted prevention programmes reaching black Africans at risk, including persons who recently travelled as well as those engaging in other more established high risk behaviours such as unprotected sex with casual, concurrent, and high numbers of, sexual partners.

Applying the 0.17% annual incidence estimate among black Africans to the 67,337 who attended sexual health clinics in 2013 equates to 115 persons with incident infections. Using a CD4 back-calculation model and date of entry into the UK, we estimate that approximately 500 black African heterosexuals acquired their infection each year in the UK [34] over the 5 years (equating to 30% of all newly diagnosed black Africans). As such, this would imply that about one in five (115/500) persons of black African ethnicity who acquire HIV in the UK each year are detected in sexual health clinics in the same year. Given that this is only a small fraction of estimated incident infections in this group, better methods are needed to track new infections in this population. Although there are no population-based incidence estimates for black Africans, in the 2011 UK census, it was estimated that there were 989628 black Africans living in England and Wales [35] (479799 men; assuming 3% are MSM (n = 14394) would approximate a heterosexual population size of 975234). Five hundred incident infections would thus approximate a population-based incidence rate of 0.05%.

For MSM, where population-based HIV incidence estimates are available, considering a 1.5% incidence among MSM attending sexual health clinics and 92037 MSM in total attended a clinic in England in 2013 [2], this would equate to 1381 MSM with incident HIV infections having attended a clinic in that year. Based on Birrel et al.’s estimates of between 2300–2500 new infections each year, this implies over half (55%) of all MSM with incident infections attend sexual health clinics and are diagnosed within a year of their infection. Community-based behavioural studies show higher risk MSM are often linked into sexual health services and are more likely to be diagnosed with a sexually transmitted infection. As such, PrEP coupled with behaviour change interventions are likely to be highly appropriate for this healthcare seeking population. Strategies to better inform and provide access to prevention strategies including PreP among other individuals at high risk of HIV infection in the UK need to be developed.
